# Isoniazid Proliposome Powders for Inhalation—Preparation, Characterization and Cell Culture Studies

**DOI:** 10.3390/ijms12074414

**Published:** 2011-07-07

**Authors:** Wipaporn Rojanarat, Narumon Changsan, Ekawat Tawithong, Sirirat Pinsuwan, Hak-Kim Chan, Teerapol Srichana

**Affiliations:** 1 Drug Delivery System Excellence Center, Department of Pharmaceutical Technology, Faculty of Pharmaceutical Sciences, Prince of Songkla University, Hat Yai, Songkhla 90112, Thailand; E-Mails: wipaporn_oil@yahoo.com (W.R.); nar_tik@hotmail.com (N.C.); ekawat281@gmail.com (E.T.); sirirat.p@psu.ac.th (S.P.); 2 Faculty of Pharmacy, University of Sydney, Sydney, NSW 2006, Australia; E-Mail: kim.chan@sydney.edu.au

**Keywords:** tuberculosis, drug delivery, immune response, liposomes

## Abstract

The aims of this study were to develop proliposome powders containing isoniazid (INH) in a dry powder aerosol form. INH-proliposome powders were prepared by a spray drying method. Proliposome physicochemical properties were determined using cascade impactor, X-ray diffraction and differential scanning calorimetry. The toxicity of proliposomes to respiratory-associated cell lines and its potential to provoke immunological responses from alveolar macrophages (AM) were determined. Free INH and INH-proliposome bioactivities were tested *in vitro* and in AM infected with *Mycobacterium bovis* (*M. bovis*). Aerosolization properties of INH-proliposome powders at 60 L/min, the powders showed mass median aerodynamic diameters of 2.99–4.92 μm, with fine particle fractions (aerosolized particles less than 4.4 μm) of 15–35%. Encapsulation of INH was 18–30%. Proliposome formulations containing INH to mannitol ratios of 4:6 and 6:4 exhibited the greatest overlapping peak between the drug and mannitol. INH-proliposomes were evidently nontoxic to respiratory-associated cells, and did not activate AM to produce inflammatory mediators—including interleukin-1β (IL-1β), tumor necrosis factor-α (TNF-α), and nitric oxide—at a toxic level. The efficacy of INH-proliposome against AM infected with *M. bovis* was significantly higher than that of free INH (*p* < 0.05). INH-proliposomes are potential candidates for an alternative tuberculosis treatment.

## 1. Introduction

Isoniazid (INH), a first-line hydrophilic antituberculosis agent, is difficult to encapsulate in liposomes which is increasingly used and the results have been very promising [1–3]. However, liposomes in aqueous systems suffer from physical instability due to hydrolysis or oxidation, resulting in the loss of the encapsulated active component from the liposome. This is especially a problem in the case of hydrophilic agents. Other potential liposome instabilities are sedimentation, and aggregation or fusion of the liposomes during storage [4–6].

Proliposome, a dry free-flowing powder, can be hydrated immediately to form liposomes through contact with water or biological fluids [5]. The purpose of this attempt is to overcome the problems associated with aqueous liposome dispersion. In general, powdered drugs are prepared by the adsorption of drugs and phospholipids onto the microporous matrix of carrier particles, typically sorbitol [4,6,7]. The microporous structure of the carrier materials maintains the free flow of the powder. The manufacturing procedures however appear to be tedious and difficult to control, since the operation requires: (a) a discontinuous step of solvent addition and evaporation; (b) close monitoring to ensure that the powder is not allowed to become overly wet, and the unit is operated under a vacuum; and (c) overnight drying of the proliposome in a desiccator under reduced pressure [8].

Spray drying has also been considered as a one-step process for the production of small particles (<5 μm) for pulmonary administration. This allows for better control of particle formation, and hence can be easily scaled up. Spray drying is not only limited to aqueous solutions, but can also be used for non-aqueous systems to prepare particles suitable for aerosol delivery [9,10]. The obtained lipid particles were spherical, with aerodynamic diameters less than 5 μm. In this study, proliposomes containing INH at various ratios of INH to mannitol were prepared. Spray-dried mannitol (3 μm) was used as a core carrier, and soybean phosphatidylcholine was coated onto its surface. Suitable mannitol-INH-phospholipid system is justified from aerosolization properties, thermal properties and degree of crystallinity. There are no systematic studies previously reported on this system. It is crucial that proliposomes reach the airways, but do not activate an immunological response from alveolar macrophages (AM). Activated AM can secrete inflammatory mediators in the presence of foreign particles [11]. In this study, we have ensured that INH-proliposomes do not cause cytotoxicity to respiratory cells. In addition, the antituberculosis efficacy of proliposomes containing INH against intracellular mycobacterial growth was also determined, and compared to that of free INH.

## 2. Results and Discussion

### 2.1. Surface and Morphology of INH-Proliposome Particles under SEM

**Figure 1:**
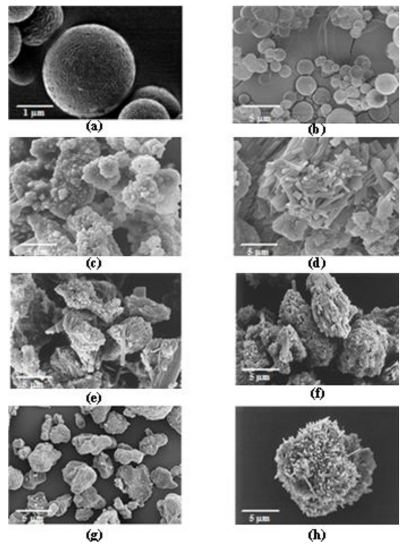
The SEM images of **(a)** spray dried mannitol (18,000×, bar = 1 μm), **(b**–**f)** INH-proliposome formulation #1 to #5 (3,700×, bar = 5 μm), **(g)** spray dried INH and **(h)** pure INH.

INH-proliposome powders were successfully prepared. Smooth spherical particles of pure mannitol with a size of around 3 μm (Figure 1a) were obtained from spray-dried mannitol at a low concentration (10 mg/mL). A large number of particles smaller than 5 μm in the aerosol could be obtained by delivering the mannitol powder using a Diskhaler^®^ [12]. Furthermore, mannitol has been previously evaluated as a potential drug carrier in dry powder inhaler formulations [13]. The surface and morphology of proliposomes obtained from formulations #1 to #5 are shown in Figure 1b–1f. Spherical mannitol microparticles were observed in formulation #1 (Figure 1b) with a high (90%) content of mannitol. Mannitol is an important ingredient to obtain spherical particles by the spray drying method. Other INH-proliposomes containing 80% mannitol and less than 80% were irregular in shape, with some tiny particles (less than 1 μm, Figure 1c) or elongated particles (Figure 1d–1f) adhering to large aggregated particles. From Figure 1g, it seems that INH by itself cannot be spray dried to produce a spherical particle. INH formulations containing mannitol less than 90% or without mannitol were irregular in shape and different from the properties of pure INH. This is affected by the spray drying process that changed formulation crystallinity and morphology. We have suggested that the ideal formulation should contain INH from no more than 10% to obtain perfect spheres.

### 2.2. Interaction of INH-Proliposome Powder and Its Physical Properties

**Figure 2:**
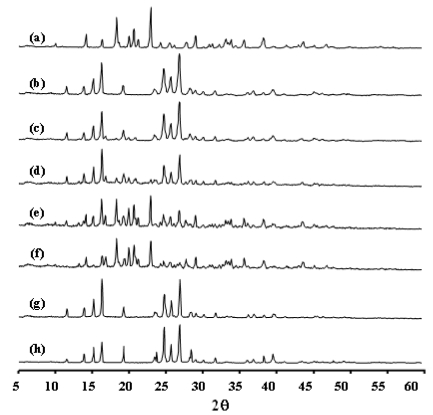
The X-ray diffraction patterns of **(a)** spray dried mannitol, **(b-f)** INH-proliposome formulation #1 to #5, **(g)** spray dried INH and **(h)** pure INH.

L-α soybean phosphatidylcholine (SPC) is an amorphous material [14] with no crystalline peak; but does have an amorphous halo. INH can exhibit some degree of crystallinity before spray drying; but after having been spray dried, INH had lower diffraction intensity (Figure 2g and 2h). This indicates that the spray drying processes might decrease the crystallinity of INH. XRD patterns of various proliposomes are shown in Figure 2b-2f; all indicate changes of the XRD pattern of INH and mannitol. This reflects a lower amount of the respective components in the formulations, and/or may indicate the interaction of INH and mannitol. Crystalline mannitol was predominant in formulations #1 and #2 (Figure 2b and 2c). However, an SPC amorphous halo did not appear on all proliposome XRD patterns. This was due to the low concentration of SPC as compared to other components. As a result, SPC has no effect on the XRD patterns of proliposomes.

**Figure 3:**
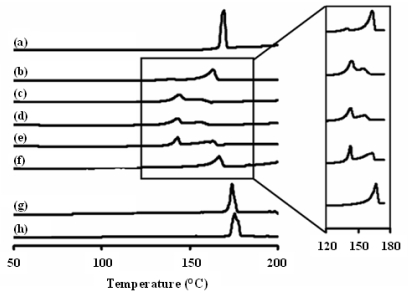
Differential scanning calorimeter thermogram of **(a)** spray dried mannitol, **(b**–**f)** INH- proliposome formulation #1 to #5, **(g)** spray dried INH and **(h)** pure INH.

From the differential scanning calorimeter (DSC) thermogram (Figure 3), the onset, end, peak temperature and enthalpy of spray-dried INH and INH-proliposome are summarized in Table 1. Spray-dried INH was in crystalline form with a lower endothermic enthalpy as compared with the starting INH. The results correlate with the obtained XRD patterns (Figure 2). For the proliposome powder, the XRD patterns of INH-lipid-mannitol were dependent on the formulation compositions. For example, formulation #5 (containing INH 80% and mannitol 20%) showed a single broad peak of 166.8 °C (7° lower than spray-dried INH). This endothermic peak was attributed to INH which was the major component. The formulation consisting of 40% mannitol (Formulation #4) showed two completely separated endothermic peaks. The first peak at 143.0 °C was related to INH-mannitol (Figure 3) while the second peak at 163 °C (3° lower than the INH-lipid peak) is expected to be from INH. The shift to the lower temperatures indicated an interaction between INH, mannitol and/or lipid. By increasing mannitol to 40% and 60% in the spray-dried formulations while decreasing the INH to 60% and 40% (Formulations #4 and #3, respectively), two incompletely separated peaks were obtained at 143.0 °C and 155.8 °C (Figure 3). However, when the INH content was as low as 20%, the two peaks were completely separated again. A small endothermic peak (140 °C) of the INH-mannitol composition was obtained. This was due to some of the INH content being lost during the spray drying process (10% INH was found in the products), resulting in the INH-mannitol composition giving only a small peak. However, its endothermic peak of 163.2 °C showed a similarity to formulation #5. The maximum interaction of INH-lipid-mannitol was found in formulations #3 and #4.

However, at higher concentrations of INH (60% or higher in the formulation), X-ray diffraction (XRD) and DSC reveal a higher degree of crystallinity of INH from the peak height and area (Figures 2 and 3) as compared to formulations #1–#3. This indicated that the loss of INH during spray drying was lower. There was no significant decrease in the INH content of formulations #3–#5 after being kept in a desiccator for 6 months (*p*-value > 0.05) (Table 2).

### 2.3. INH Recovery, Encapsulation Efficiency (EE) and Size after Reconstitution of INH Proliposome into a Liposome Suspension

The INH contents showed a slight decrease from the initial amounts. From Table 1, the uniformity of drug content was acceptable (%RSD less than 6, USP 30 and NF 25; USP-NF, 2007). The spray drying process is able to produce a uniform distribution of the active ingredient throughout the proliposome product. However, the percentage of recovered INH content in formulations #1 to #3 was less than the INH in formulations #4 and #5. From this observation, when the INH content was lower than the mannitol content in the formulation, a significant loss of INH was clearly observed. When INH content was only 10–40%, it may exist partially in an amorphous state. A lower diffraction intensity peak in the 2θ range of 23–30° was obtained from formulations #1 and #2, indicating a lower crystallinity of INH from the peak height and area under the curve. When such a formulation was processed in a spray dryer, the majority of INH was lost by melting and by deposition on the drying chamber of the equipment, resulting in a low percentage recovery.

After reconstitution of the INH-proliposomes, vesicle sizes (Table 2) exhibited a normal distribution. The mean size ranges were 329–461 nm from formulations #1, #2 and #5, while liposome vesicle sizes of formulations #3 and #4 were larger (approximately 700–1000 nm). The decreasing in size of formulations was due to solubilization of mannitol. This was confirmed by atomic force microscopy (AFM) analysis of rehydrated proliposomes. Although these mean size ranges of all formulations (300–1000 nm) are in the nanoscale, it is much higher than typical nanovesicles (200 nm). Previous studies reported nanoparticles of 200 nm in size were efficiently taken up by macrophage [15,16]. In fact when the mean size was 300–400 nm, the size distributions were in ranges of 100 to 500 nm. It indicates that some nanovesicles will be available for AM uptake and larger particles are for extracellular killing. The surface of liposome is also important factor that cells response differently to different surfaces. However, in this case the liposome particles look very similar in their surface.

The encapsulation efficiency of INH was in the range of 18–30% (Table 2). In this study, the INH-proliposome was reconstituted with distilled water, whereas free INH would dissolve instantly. The INH-encapsulated liposome was separated by ultracentrifugation. The supernatant containing free INH was analyzed for its INH content. Formulations #1 and #2 had the highest INH encapsulation, while formulation #5 had the lowest. This indicated that a high INH loading in the formulation resulted in a low amount of encapsulated INH. Those formulations with a low INH loading had high encapsulation efficiency. INH could be incorporated into mannitol particles before the lipid coating. As a result, when the INH loading was high, it was impossible for the lipid to coat all the INH particles. An INH:mannitol ratio of 1:9 seemed to give the highest encapsulation efficiency.

### 2.4. Aerosol Performance of Proliposome Powder

**Figure 4:**
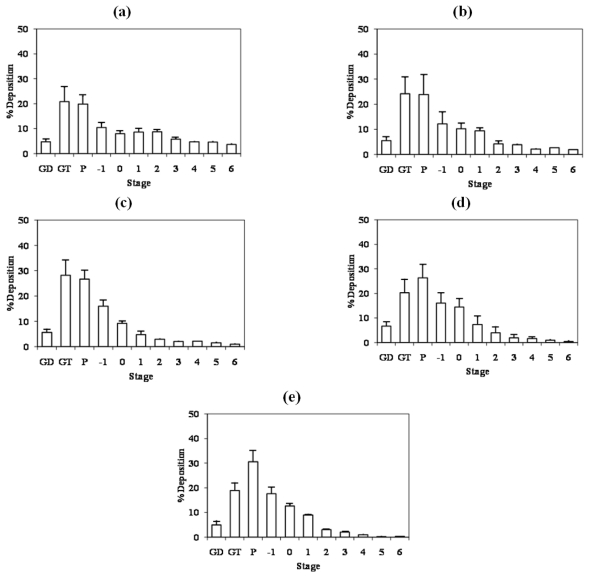
The particle size distribution of **(a-e)** Isoniazid (INH)-proliposome formulations #1–5 after aerosolization into the Andersen Cascade Impactor at flow rate of 60 L/min for 10 seconds (GD = Glass Device, GT = Glass Throat, P = Preseparator) (mean ± SD, n = 3).

The particle size distribution of INH-proliposome powder is shown in Figure 4. INH-particles deposited on glass device (GD), glass throat (GT) and preseparator (P) while other fractions deposited on stages −1 to 6. INH-proliposomes containing 10% INH (Formulation #1) produced by the spray drying technique generated a significantly smaller MMAD than did other INH proliposomes (Formulation #3–#5) (2.99 μm, *p* < 0.01). Other proliposome formulations (40–80% INH) showed no difference in mass median aerodynamic diameter (MMAD) (4.46–4.92 μm, *p* > 0.05). MMAD values were correlated with a low fine particle fraction (FPF) of 15%. The lower MMAD of formulation #1 gave a higher FPF. An ED of 91–95% was obtained from the formulations, with the highest value of 95% found in formulation #1. The MMAD should be less than 5 μm in order to reach the lower part of the respiratory tract, and all prepared formulations met this requirement. Also, it is important to note that the MMAD and FPF will also depend on the inhaler device. The use of microparticulate mannitol as a core carrier, in this case, improved the aerosolization characteristics of the INH-proliposome powder; but the %FPF was low. It might be a result from the agglomeration of microparticulate mannitol coated with SPC. SPC was mainly deposited on the surfaces of the mannitol microparticles, and this enhanced particle agglomeration [5]. In order to overcome this problem, microporous particles could be used instead of smooth-surface microparticles. This type of particle would allow the lipids to be deposited in the porous structure, thus rendering less lipid surface contact with other particles and resulting in lower agglomeration [5]. Using microporous particles, more lipid content could be used in order to maximize encapsulation. In addition, microporous particles have a high void space, and may improve aerosol performance [17]. Also, the optimum amount of the drug content is a crucial factor to be considered.

### 2.5. Viability of Respiratory Cell Lines after Exposure to INH-Proliposomes

**Figure 5:**
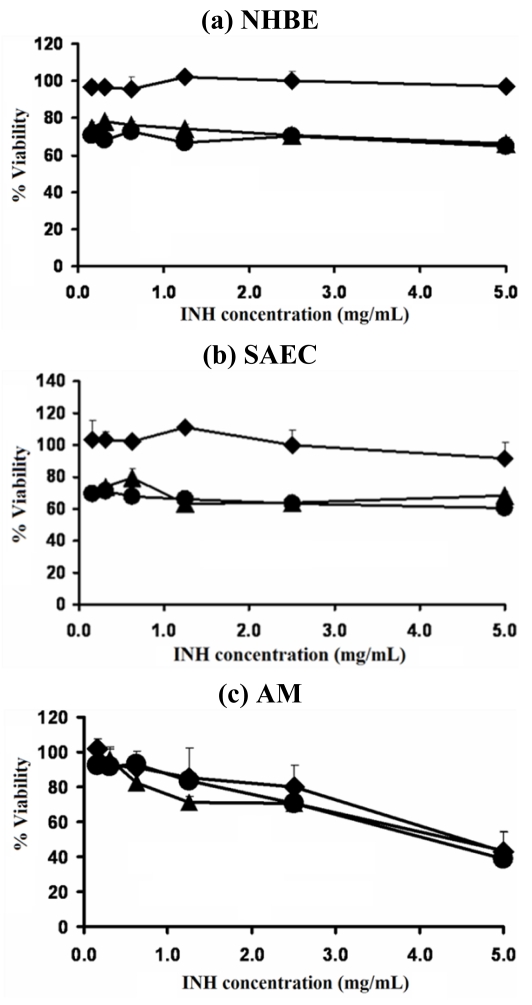
Viability of **(a)** NHBE, **(b)** SAEC and **(c)** AM cell lines after exposed with different concentrations of free INH (●), INH-proliposome formulation #1 (♦) and INH proliposome formulation #5 (▴) (mean ± SD, n ≥ 6).

The viabilities of normal human bronchial epithelial cells (NHBE), small airway epithelial cells (SAEC) and AM were estimated after being challenged with different concentrations of free INH and INH-proliposomes (Formulation #1 and #5). Formulations #1 and #5 were the selected formulations because their mannitol content varied from the highest to the lowest. The effect of INH, in different amounts of mannitol in the formulation was established on respiratory cell lines. INH-proliposome formulation (#1) was not toxic to respiratory cell lines at concentrations of less than 2.5 mg/mL (Figure 5a and 5b), with almost 100% viability of AM (Figure 5c). The viability of AM cells decreased at higher concentrations (2.5 and 5 mg/mL); for example, only 40% viability was obtained for the INH concentration of 5 mg/mL. The % viability of AM was not significantly different between free INH and INH-proliposome (*p* > 0.05). However, for the respiratory epithelial cell lines both NHBE and SAEC were likely to be more sensitive to INH-induced toxicity. All concentrations of free INH and INH-proliposome formulation #5 reduced the viability of NHBE and SAEC to 70%. Viabilities were reduced from the INH burst release in liposome formulation #5. As the drug loading is much higher than other formulations, initial burst release was found to occur in the first hour over 40% of INH. A viability of 100% was obtained with INH-proliposome formulation #1, which had the lowest INH content (10%). It was clearly indicated that mannitol was not toxic to respiratory cell lines. However, the different amount of mannitol at the same concentration of INH did affect the viability of respiratory cell lines. This may be due to a stabilizing effect of this sugar.

As will be discussed later, the toxic concentration of 2.5 mg/mL (which is unlikely to occur in the respiratory tract following administration by inhalation) is 5,000 times higher than the minimum inhibitory concentrations (MIC) of INH against the TB bacillus.

### 2.6. Effect of INH Proliposome on Production of IL-1β, TNF- α and Nitric Oxide by AM

The AM cells exhibited characteristics of macrophage cells: phagocytosis of zymosan and *Pseudomonas aeruginosa*; nonspecific esterase activity; Fc receptors; oxidative burst; IL-1, TNF-α and IL-6 secretion; and replicative response to exogenous growth factors. The cells responded to particulate or soluble stimuli by phagocytosis and killing. The NR8383 cell line provides a homogenous source of highly responsive alveolar macrophages that can be used *in vitro* to study macrophage-related activities [18]. Hence, liposomes may be recognized by AM and activated AM produce an immunological response. For safety reasons, the level of inflammatory cytokines was examined from the cultured supernatant of AM after exposure to proliposomes for 24 h.

**Figure 6:**
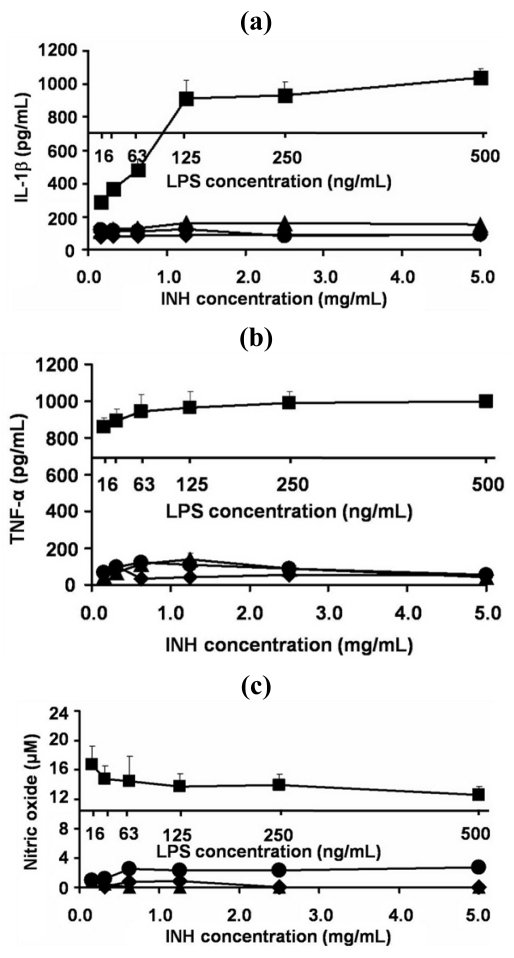
The level of inflammatory cytokine (**(a)** IL-1β, **(b)** TNF-α) and **(c)** nitric oxide produced from AM cell lines after exposure with different concentrations of free INH (●), INH-proliposome formulation #1 (♦), INH proliposome formulation #5 (▴) and LPS from *E. coli* (■) for 24 h (mean ± SD, n ≥ 6).

As expected, AM produced lower amounts of IL-1β, TNF-α and nitric oxide in response to INH-proliposome and free INH, compared to the LPS (lipopolysaccharide) from *E. coli* (Figure 6a, 6b and 6c, respectively). The concentration of LPS used to stimulate AM to produce the immunological response was 10^4^ times less than the concentration of any INH-proliposome. LPS activation of AM to produce inflammatory cytokines was significantly greater than when it was challenged with INH-proliposomes (*p* < 0.05).

### 2.7. Activity of INH and INH-Proliposome Against M. bovis

**Figure 7:**
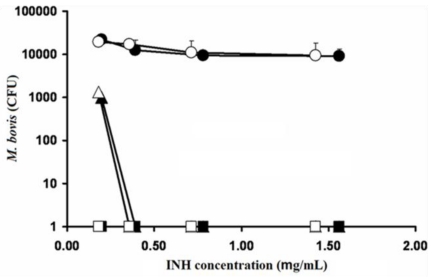
The reduction of *M. bovis* (CFU) after incubated with free INH (dark symbol) and INH-proliposome formulation #5 (blank symbol) at day 1 (●,○), day 3 (▴,▵) and day 7 (■,□) (Mean ± SD, n ≥ 3).

The remaining colony-forming units (CFU) of *M. bovis* after incubation with INH or INH-proliposome at various concentrations are shown in Figure 7. INH-proliposome formulation #5 which had the lowest %EE (Table 2) was chosen for INH activity testing against *M. bovis* and *M. tuberculosis*. This formulation was chosen based on the stability data after 6 months storage, mean size and flexibility in dose adjustment from highest INH loading. Free INH and INH-proliposome showed similar activity against *M. bovis*. At day 1, the drug showed no effect against *M. bovis* at all concentrations. However, the CFU were completely cleared with INH concentrations of 0.4 μg/mL or higher at day 3 (both free INH and INH-proliposome). In addition, at day 7 there were no viable cells when *M. bovis* was challenged with all concentrations of INH and INH-proliposome. These results demonstrated that INH and INH-proliposomes were able to kill the bacilli by the third day of incubation, and were even more effective on day 7. The MIC of INH and INH-proliposome for day 3 was 0.4 μg/mL, while the MIC for day 7 was less than 0.2 μg/mL. From this result, it is indicated that INH activity against *M. bovis* of pure INH and INH formulation showed similar activity even though the lowest %EE formulation was employed.

### 2.8. Activity of INH and INH-Proliposome Against M. tuberculosis

The same MIC values of free INH and INH-proliposome formulation #5 against *M. tuberculosis* were obtained at a concentration of 0.024 μg/mL. This indicated that INH and INH-proliposome were able to kill the bacilli at a concentration twice as low as that reported by Mohamad and co-workers (2004) [19], and 16.7 times lower than that reported by Chanwong and co-workers (2007) [20]. However, the most important point is that in our experiments both free INH and INH-proliposome exhibited a similar efficacy against the TB bacillus. This is in accordance with the burst release of INH from INH proliposome which gave high free INH viability.

### 2.9. Activity of INH and INH-Proliposome on Intracellular Growth of M. bovis

The activity of free INH and INH-proliposome formulation #5 against intracellular infection of AM by *M. bovis* was examined at various incubation times and concentrations. From the results shown previously, the MIC of INH against *M. bovis* at day 3 was 0.4 μg/mL. Concentrations of 12.5, 25 and 37.5 times the MIC were used to treat AM -infected *M. bovis*. Based on such calculations, the final concentration of INH (or the equivalent in the case of INH-proliposome formulations) would be 5, 10 and 15 μg/mL in the cell culture media.

**Figure 8:**
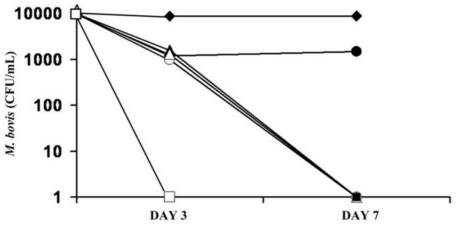
Activity of free INH (dark symbol) and INH-proliposome formulation #5 (blank symbol) against intracellular growth of *M. bovis* at concentrations of 5 μg/mL (●,○), 10 μg/mL (▴,▵), 15 μg/mL (■,□) and control (♦) (Mean ± SD, n ≥ 3).

As shown in Figure 8, at the highest concentration of 15 μg/mL, the CFU were completely cleared by both INH and INH-proliposome at day 3 and day 7. There was no significant difference between the forms of INH (free or INH-proliposome) at this high concentration. At 10 μg/mL, the bacilli were completely killed by day 7, again with no significant difference between formulations (*p* > 0.05). At a concentration of 5 μg/mL, free INH was ineffective in killing bacilli at either day 3 or day 7, whereas INH-proliposome showed antimycobacterial activity against intracellular *M. bovis* after 7 days of incubation. This may be due to the hydrophilic properties of INH that makes it difficult to penetrate the AM. However the incorporation of INH with liposome enhanced permeation, thus attaining a higher intracellular concentration. Hence, high activity against *M. bovis* in AM was obtained. In clinical use, INH-proliposome might show good clinical outcomes.

## 3. Experimental Section

### 3.1. Materials

Mannitol, L-α soybean phosphatidylcholine (SPC), and cholesterol from lanolin (CH) were obtained from Fluka (Buchs, Switzerland). INH, sulfanilamide and *N*-(1-naphthyl)-ethylenediamine dihydrochloride (NED) were from Sigma-Aldrich (St. Louis, MO, USA), and dimethyl sulfoxide (DMSO) from Riedel-de Haën (Seelze, Germany).

All solutions (reagent packs) used for maintenance and culture of normal human bronchial epithelial cells and small airway epithelial cells were acquired from Lonza Group, Ltd. (Walkerville, MD, USA). F-12 Kaighn’s cell culture medium with 2 mM l-glutamine was adjusted to contain 1.5 g/L sodium bicarbonate (Gibco, Grand Island, NY, USA), supplemented with 15% (v/v) heat-inactivated fetal bovine serum, 50 units/mL of penicillin, and 50 μg/mL of streptomycin (Gibco). A BEGM BulletKit^®^ and a SAGM BulletKit^®^ were from Lonza Group, Ltd. A sample of 3-(4,5-dimethylthiazol-2-yl)-2,5- diphenyltetrazolium bromide (MTT) was from Sigma-Aldrich. Dulbecco’s phosphate buffered saline (DPBS) was from Gibco. Quantikine^®^ RTA00 and Quantikine^®^ RLB00 kits for rat TNF-α and IL-1β, respectively, were from R&D Systems Inc. (Minneapolis, MN, USA). The BCG vaccine of *Mycobacterium bovis* was supplied by Aventis Pasteur (Paris, France). Middlebrook 7H9 culture medium was from Becton Dickinson and Co. (Franklin Lakes, NJ, USA). *M. tuberculosis* H_37_Ra (ATCC 25177) cells from the American Type Culture Collection (Rockville, MD, USA). Alamar blue solution was from Alamar Biosciences/Accumed (Westlake, OH, USA).

### 3.2. Production of Microparticulated Mannitol

A solution of mannitol in distilled water (10 mg/mL) was sprayed through a 0.7 mm nozzle using a B-191 spray dryer (Büchi, Flawil, Switzerland) at an inlet temperature of 110 °C, an atomizing pressure of 800 kPa, and a feeding rate of 3 mL/min. The outlet temperature was 90 ± 2 °C. The product was separated and collected by the cyclone and then directed into the collecting chamber. The obtained microparticles were used as core carriers of the proliposome preparations.

### 3.3. Production of INH-Proliposome by Spray-Drying Technique

The ingredients of the proliposome formulations are shown in Table 1. Briefly, microparticulate mannitol (particle size ~3 μm) was used as a core carrier. A lipid solution containing INH and microparticulate mannitol was spray dried to obtain the proliposomes.

**Figure 9:**
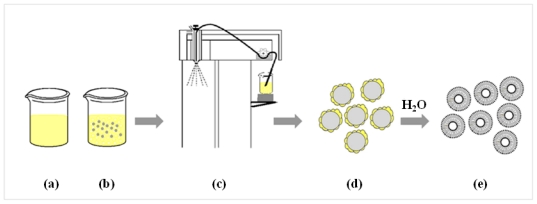
The scheme of proliposome production by spray drying technique. **(a)** ethanolic lipid solution containing INH; **(b)** dispersion of fine mannitol in ethanolic lipid solution containing INH; **(c)** spray drying processes; **(d)** proliposome powder product; and **(e)** reconstituted proliposome powder.

Lipids were composed of a mixture of l-α soybean phosphatidylcholine (SPC) and cholesterol from lanolin (CH) in a mole ratio of 1:1. These were weighed and dissolved in 100 mL of 95% ethanol to obtain an ethanolic lipid solution. INH was added to the ethanolic lipid solution and sonicated until a clear solution was obtained (Figure 9a). The maximum concentration of INH in this study was only 1%, resulting in the INH being completely dissolved (The solubility of INH in ethanol is 2%). Microparticulate mannitol was dispersed in the solution, and the suspension was sonicated for 15 min in order to deaggregate mannitol particles before the spray drying process began (Figure 9b). The suspension was continuously stirred to provide homogeneity of the suspension during spray drying (Figure 9c). The inlet temperature was 90 °C and atomizer pressure was 800 KPa, with a feed rate of 3 mL/min. The outlet temperature was 70 ± 1 °C. The proliposome powder was transferred from the collecting chamber into a desiccator until used.

### 3.4. Scanning Electron Microscopy of the Proliposome Powders

The surface morphology of the proliposome particles was examined by scanning electron microscopy (SEM). A sample was sprinkled onto an aluminum stub and coated with gold by a sputtering technique using a JFC-1200 Fine Coater (JEOL, Tokyo, Japan) for 120 s. The particles were observed under SEM (JSM-6301F, JEOL) at 3 kV.

### 3.5. Content Uniformity of INH in the Proliposome Powder

INH-proliposome powder (10 mg) was randomly sampled and weighed. The powder was suspended in 10 mL of methanol to dissolve the lipid content. The volume was adjusted to 25 mL with distilled water, followed by sonication to obtain a clear solution. The INH content from the clear solution was analyzed by high performance liquid chromatography (HPLC). The HPLC system was equipped with an AS 3000 autosampler, a P1000 pump and a UV 2000 detector (Thermo Fisher Scientific, Waltham, MA, USA). The mobile phase was phosphate buffer (0.2 M):acetonitrile (97:3 v/v), running at a flow rate of 1 mL/min. UV detection was at 254 nm. A microbondapak C18 column (Phenomenex, Torrance, CA, USA) (250 × 4 mm i.d., 5 μm) was used as a stationary phase.

### 3.6. Encapsulation and Size Measurements after Reconstitution of INH Proliposome into a Liposome Suspension

The INH-proliposome powder (10 mg) was weighed and reconstituted with 1 mL of 0.2 M phosphate buffer solution (PBS), pH 7.4, and then incubated for 10 min to form a liposome suspension. The reconstituted suspension (100 μL) was transferred to a 10 mL volumetric flask. Methanol (2 mL) was added to dissolve the lipid coating on the particles. The solution was then adjusted to the final volume with deionized water and analyzed by HPLC for its INH content to determine the total drug loading.

To determine the percentage encapsulation, 10 mg of proliposome powder was reconstituted with distilled water (4 mL), and then centrifuged with a SW 60 Ti rotor (Beckman Coulter Inc., Palo Alto, CA). The centrifugation conditions were 100,000 *g* for 20 min at 25 °C [21]. The supernatant was analyzed as unencapsulated INH by HPLC, as described in the content uniformity section. The % encapsulation efficiency (%EE) was obtained using the following equation. (Total drug loading unencapsulated drug)

$$\%\text{EE}=\frac{\left(\text{Total drug loading}-\text{unencapsulated drug}\right)}{\text{Total drug loading}}\times 100$$

The size of the liposome after reconstitution was measured using ZetaPALS (Brookhaven, NY, USA) at 25 °C. The proliposome powder was reconstituted with milliQ water to obtain nanovesicles while the undissolved particles were removed by centrifugation. The centrifugation conditions were 10,000 g for 20 min at 25 °C. Sizes were determined immediately after obtaining the supernatant from the centrifugation.

### 3.7. X-ray Diffraction Measurement of INH-Proliposome

X-ray diffraction (XRD) of spray-dried mannitol, INH and INH-proliposome was carried out with a Siemens D 5000 (Siemens AG, Berlin, Germany) equipped with a diffracted-beam monochromator, using Cu radiation. The proliposome powder samples were spread on glass sample holders, each in an area of 4 cm^2^ with a depth of 1 mm. The powder surfaces were pressed and smoothed with a glass slide. Diffraction intensity was recorded at an angle of 2θ from 5 to 60° with a step size of 0.05° and step time of 1 s. The total time of the diffraction scan was 19 min, and each sample was examined in three separate experiments. The voltage and current generator were set at 40 kV and 30 mA, respectively. The obtained data were analyzed by EVA software. Relative crystallinity was determined from the XRD results by the ratio of the intensity of a characteristic crystalline peak to that of the amorphous halo for each powder sample. If the ratio of the peak to the amorphous halo around it decreases, then the crystallinity is proportionately lower [22].

### 3.8. Differential Scanning Calorimetry of INH Proliposome

A differential scanning calorimeter (DSC) model 2920 (TA Instruments, Newcastle, DE, USA) was used to investigate the interaction of INH-mannitol-SPC in the proliposome powder produced by spray drying. A powder sample was placed in an aluminum pan, hermetically sealed, and then assessed by DSC from 50 °C to +200 °C at a rate of 10 °C/min. The DSC thermograms were analyzed using the Universal Analysis 2000 program, version 3.4c.

### 3.9. *In Vitro* Evaluation of Aerosol Performance of the INH-Proliposome Dry Powder by a Cascade Impactor

The INH-proliposome dry powder was aerosolized using a dispersion device made in-house [23]. The aerosolized parameters of the products—including mass median aerodynamic diameter (MMAD), fine particle fraction (FPF) and emitted dose (ED)—were evaluated using an Andersen cascade impactor (ACI) (Andersen Instruments, Atlanta, GA, USA). The ACI was applied with a vacuum pump under a flow rate of 60 L/min for 10 s. Triton X-100 (0.1%) in methanol was used to rinse particles deposited on each stage. The INH content was determined by HPLC, as described in the content uniformity section. The cumulative percentage of deposition was transformed to Z-values and plotted against the log cutoff diameter of each stage. MMAD is obtained from the particle diameter at a Z-value of zero [24]. The emitted dose is the amount of drug propelled from the delivery device. FPF is calculated from the amount of drug deposited on stages 1–6, which is the fraction of particles smaller than 4.4 μm.

### 3.10. Cell Cultures

#### 3.10.1. Growth of Normal Human Bronchial Epithelial Cells (NHBE) and Small Airway Epithelial Cells (SAEC)

Normal human bronchial epithelial cells (NHBE) and small airway epithelial cells (SAEC) were cultured in Clonetics^®^ media supplemented with either a BEGM or a SAGM bullet kit^®^. The cells were cultured by following the protocol recommended by Lonza. Briefly, the cells were seeded at a density of 3,500 cells/cm^2^ for NHBE and 2,500 cells/cm^2^ for SAEC. The cells were incubated at 37 °C, 5% CO_2_ and 95% humidity. When the cells reached 60–80% confluency, they were rinsed with HEPES buffered saline solution (HEPES-BSS) to wash off any remaining complex proteins that might neutralize the trypsin activity. HEPES-BSS was aspirated, and the cells were covered with 2 mL of trypsin/EDTA solution. The cells were then detached from the plate by the trypsin/EDTA; trypsin activity was then neutralized with trypsin neutralizing solution. Cells were centrifuged, resuspended and then transferred to a new culture flask. The cells were used in early passage numbers (8–10), as recommended by Clonetics.

#### 3.10.2. Alveolar Macrophage Cell Line NR8383 (AM)

A rat alveolar macrophage cell line NR8383 (ATCC CRL-2192, Rockville, MD, USA) had been isolated from normal rat lung lavage. The cells were cultured in F-12 Kaighn’s cell culture medium with 2 mM l-glutamine supplemented with 15% (v/v) heat-inactivated fetal bovine serum, 50 units/mL penicillin, 50 μg/mL of streptomycin, and then incubated at 37 °C, 5% CO_2_ and 95% humidity. Cultures were maintained by transferring floating cells to additional flasks. Adherent cells may be harvested by scraping. Upon reseeding, about half of the cells re-attached. The medium was replaced with fresh medium two or three times weekly.

#### 3.10.3. Determination of Cytotoxicity of INH-Proliposome to Cells in the Respiratory Tract

Viabilities of NHBE, SAEC and AM were determined using the MTT assay after exposure to both free INH and INH-proliposomes in a concentration range of 0.15–5.0 mg/mL. Live mitochondria transform MTT into formazan, which can be measured with a spectrophotometer. Briefly, 100 μL of 1 × 10^5^ cells/mL was cultured in each well of a 96-well plate and allowed to attach and grow overnight at 37 °C, 5% CO_2_ and 95% humidity. The following day, the medium (100 μL) was replaced with media containing either INH-proliposome or free INH solution (100 μL). The plate was then incubated for 24 h. The supernatant from each well of the AM culture was removed to examine the level of generated inflammatory cytokines by the ELISA method or of nitric oxide by the Griess reagent, as described in the determination of alveolar macrophage response section. Viable cells were quantified and compared with the untreated control by the MTT assay. Filtered sterilized stock MTT solution (50 μL of 5 mg/mL in Dulbecco’s phosphate buffered saline, DPBS) was added into each well containing 150 μL fresh medium, and then incubated for 4 h at 37 °C. After that, the supernatant was carefully removed, and the resulting formazan crystals were dissolved by adding 200 μL of dimethyl sulfoxide (DMSO) and mixing thoroughly. The absorbance was recorded at 570 nm with a microplate reader (Biohit BP 800, Helsinki, Finland). The proportion of viable cells in the treated wells was compared to the untreated wells [25,26]. Lipopolysaccharide (LPS) from *E. coli* was used as a positive control.

### 3.11. Determination of the Alveolar Macrophage Response to INH-Proliposome

#### 3.11.1. Production of Inflammatory Cytokines

The TNF-α and IL-1β produced from AM after challenging with free INH, INH-proliposome and LPS (positive control) were analyzed from the cell culture supernatant immunochemically with commercial enzyme-linked immunosorbent assay (ELISA) kits (Quantikine^®^ RTA 00 and Quantikine^®^ RLB 00 for rat TNF-α and IL-1β, respectively), as described in the ELISA assay procedures. TNF-α or IL-1β was calculated from a standard curve by measuring UV absorbance at 570 nm. The detectable dose of both TNF-α and IL-β was in range of 12.5–400 pg/mL.

#### 3.11.2. Nitric Oxide Assay by the Griess Reaction

Nitric oxide (NO) released by the AM after challenging with free INH or INH-proliposome in a concentration range of 0.15–5.0 mg/mL was detected by the Griess reaction. This method was used to investigate nitric oxide in the form of nitrite (NO_2_^−^), which is one of the two primary, stable and nonvolatile products of NO. This measurement relies on a diazotization reaction of the Griess reagent. Griess reagent was prepared by mixing 1% sulfanilamide, 0.1% *N*-(1-naphthyl)-ethylenediamine dihydrochloride and 2.5% phosphoric acid in water. Equal volumes of cell supernatant (100 μL) and Griess reagent (100 μL) were mixed. After mixing for 10 min, the absorbance was determined using a microplate reader at 450 nm. The NO concentration was calculated from a sodium nitrite standard curve [25,26].

### 3.12. Assessments of the Antimycobacterial Activity of Free INH and INH-Proliposome

#### 3.12.1. Culture of *M. bovis* from BCG Vaccine

The lyophilized BCG vaccine of *M.bovis* was reconstituted with 1 mL of sterile deionized water for injection. Reconstituted BCG vaccine (200 μL) was grown in Middlebrook 7H9 containing 0.5% Tween 80 and 10% oleic acid-albumin-dextrosecatalase (OADC) enrichment [27]. The bacilli were incubated at 37 °C and subcultured every 3 weeks. The obtained *M. bovis* suspension at 3 weeks old was used in this experiment.

#### 3.12.2. Determination of Minimum Inhibitory Concentration against *M. bovis*

The minimum inhibitory concentrations (MIC) of free INH and INH-proliposome (Formulation #5) in a drug concentration range of 0.2–1.6 μg/mL were determined. An inoculum of *M. bovis* was prepared from a suspension of 3-week-old organisms in M7H9 broth supplemented with Middlebrook OADC enrichment. The suspension of bacilli was adjusted with normal saline solution to obtain a turbidity of a McFarland standard of 1. Tenfold serial dilution (10^1^, 10^2^, 10^3^, and 10^4^) of the above inoculum suspension was made in normal saline solution. Each proliposome concentration (100 μL) was added into wells containing the diluted bacilli suspension (100 μL), and incubated at 37 °C. At days 1, 3 and 7, 10 μL of sample was taken from each well, dropped on a M7H9 agar plate (M7H9 broth with 1.5% w/v agar powder), and then incubated at 37 °C for 3 weeks. Each experiment was done in triplicate. After 3 weeks, the colonies were counted using a colony counting machine (Suntex Instruments Co., Ltd., Taipei, Taiwan).

#### 3.12.3. Determination of the Minimum Inhibitory Concentration against *M. tuberculosis*

The antimycobacterial activity of INH-proliposome (Formulation #5) against *M. tuberculosis* H37Ra (ATCC 25177) was determined. *M. tuberculosis* was grown in 100 mL M7H9 broth supplemented with 10% OADC enrichment and 0.05% Tween 80. Antimicrobial susceptibility testing was performed in a dark room using clear-bottomed, 96-well microplates in order to minimize background fluorescence. The outer wells of the microplates were filled with sterile water to prevent dehydration in the experimental wells. First samples were diluted with distilled deionized water; subsequent two fold dilutions were performed in 0.1 mL of M7H9 (without Tween 80). The final colony-forming unit (CFU) bacterial density of 5 × 10^4^ CFU/mL was loaded into the wells. Wells containing sample only were used to detect the autofluorescense of compounds. Plates were incubated at 37 °C. After 4 days of incubation, 20 μL of 10× Alamar Blue solution (Alamar Biosciences/Accumed, Westlake, OH, USA) and 12.5 μL of 20% Tween 80 were added to either test wells (containing bacilli) or control wells, and plates were incubated at 37 °C. A color change from blue to pink was observed at 12 and 24 h. If a well containing bacteria became pink by 24 h, reagent was added to the entire plate. If the well remained blue, additional bacteria was loaded and tested daily until a color change occurred, at which time reagents were added to all remaining wells. Plates were then incubated at 37 °C, and results were recorded at 24 h post-reagent addition. Visual MICs were defined as the lowest concentration of drug that prevented a color change [28].

#### 3.12.4. Determination of Intracellular Antimycobacterial Activity against *M. bovis*

The antimycobacterial effectiveness of INH-proliposome and free INH against intracellular growth of *M. bovis* in AM was examined at relative INH concentrations of 5, 10 and 15 μg/mL. Prior to infection, AM cells were plated at a concentration of 10^5^ cells/well in 12-well tissue culture plates, and incubated overnight in a CO_2_ incubator to allow the cells to adhere to the well surface. Fresh medium containing 1% fetal bovine serum (FBS) was used as a replacement in order to reduce cell proliferation; on the following day the penicillin-streptomycin mixture was excluded to avoid interference by the antibiotics. *M. bovis* was suspended in F-12 Kaighn’s medium containing 1% FBS, and the suspension was dispersed into individual wells at a density of 5 mycobacterium per macrophage. Infected AM were then incubated at 37°C, 5% CO_2_ for 4 h. Following incubation, the supernatant was aspirated and the wells were washed by 3 × 1000 μL with DPBS to remove unphagocytosed mycobacteria [29]. Fresh medium with or without sample was added into each well. The well plates were incubated for either 3 or 7 days. After the specified time, the medium was discarded and the wells were carefully washed three times with DPBS to remove the excess sample. Determination of CFU was conducted by lysing attached AM with 400 μL of 0.125% sodium dodecyl sulfate (SDS) in DPBS (w/v), and incubating at 37 °C for 15 min. After that, M7H9 broth (600 μL) was added into each well to make a total volume of 1 mL. The solution from each well (100 μL) was taken to dilute serially with M7H9 broth. Samples (10 μL) from each dilution were taken to drop onto the surface of M7H9 agar plates, and incubated at 37 °C for 3 weeks. Each sample was done in triplicate. After 3 weeks, the numbers of CFU were enumerated.

### 3.13. Statistical Analysis

Data, when applicable, are presented as mean ± standard deviation (SD) from at least three samples unless indicated. The data were compared using Student’s *t* test for independent samples and by analysis of variance (ANOVA) with Tukey’s multiple group comparison procedure. All statistical comparisons were calculated using SPSS software version 16.0 (SPSS Inc., Chicago, IL). A *p*-value <0.05 was considered statistically significant.

## 4. Conclusions

INH-proliposome powders were produced by spray drying. Depending on the ratios of INH to mannitol, the powders reflected the crystalline nature of the respective components and varying degrees of interaction with the lipid present in the formulations. Dispersion of the powders showed MMADs in a range of 2.99–4.92 μm, with a FPF of 15–35%. INH and INH-proliposomes were shown to be nontoxic to respiratory-associated cells, and also did not activate AM to produce inflammatory cytokines and nitric oxide at a level that would cascade to a secondary inflammation. Most importantly, INH-proliposome exhibited better antimycobacterial activity against *M. bovis*-infected AM than did free INH.

## Figures and Tables

**Table 1:** Differential scanning calorimeter data of spray dried mannitol, isoniazid (INH)-proliposome formulations, spray dried INH and pure INH. Soy phosphatidyl choline (SPC) and cholesterol (CH).

Material or formulation	Lipid part		Powder part		Peak (°C)	Onset (°C)	End (°C)	Peak area (J/g)

	SPC (mmole)	CH (mmole)	Mannitol (g)	INH (g)				
**spray dried mannitol**					169.5	166.9	171.0	283.4
**#1**	0.06	0.06	0.9	0.1	140.0	134.0	142.5	15.6
					163.2	157.4	165.6	171.0
**#2**	0.06	0.06	0.8	0.2	143.5	138.6	161.1	208.7
					154.8			
**#3**	0.06	0.06	0.6	0.4	143.0	137.2	160.8	192.6
					155.8			
**#4**	0.06	0.06	0.4	0.6	143.0	139.8	144.8	61.1
					163.0	149.7	165.3	82.3
**#5**	0.06	0.06	0.2	0.8	166.8	161.8	169.3	169.6
**spray dried INH**					174.0	172.0	176.4	218.9
**pure INH**					175.7	173.0	178.8	223.1

**Table 2:** Isoniazid (INH)-proliposome formulations containing INH and the aerosolization properties (mean ± SD, n = 3). Encapsulation efficiency (EE), emitted dose (ED), fine particle fraction (FPF) and mass median aerodynamic diameter (MMAD).

Formulation	Recovery (%)	Content uniformity (%)	Content after 6 month storage(%)	%EE	%ED	%FPF	MMAD (μm)	Mean size after reconstitution (nm)
**#1**	71 ± 2	102 ± 3	94 ± 1	30 ± 1	95 ± 2	35 ± 4	2.99 ± 0.4	339 ± 10
**#2**	72 ± 2	102 ± 2	91 ± 1	29 ± 3	92 ± 4	24 ± 2	3.58 ± 0.4	442 ± 19
**#3**	71 ± 3	101 ± 4	104 ± 1	24 ± 1	92 ± 3	15 ± 1	4.92 ± 0.9	717 ± 28
**#4**	82 ± 2	102 ± 3	103 ± 1	17 ± 2	93 ± 2	16 ± 4	4.46 ± 0.5	1093 ± 105
**#5**	81 ± 1	101 ± 1	102 ± 2	18 ± 1	91 ± 3	15 ± 1	4.79 ± 0.9	445 ± 14
